# Bifico relieves irritable bowel syndrome by regulating gut microbiota dysbiosis and inflammatory cytokines

**DOI:** 10.1007/s00394-022-02958-0

**Published:** 2022-08-02

**Authors:** Yanlin Zhou, Fan Zhang, Liqi Mao, Tongfei Feng, Kaijie Wang, Maosheng Xu, Bin Lv, Xi Wang

**Affiliations:** 1 Department of Gastroenterology, The First Affiliated Hospital of Zhejiang Chinese Medical University, Hangzhou, 310003 Zhejiang China; 2 The First Clinical College of Zhejiang Chinese Medical University, Hangzhou, 310053 Zhejiang China; 3 Key Laboratory of Digestive Pathophysiology of Zhejiang Province, The First Affiliated Hospital of Zhejiang Chinese Medical University, Zhejiang Chinese Medical University, Hubin Campus, Hangzhou, 310006 China; 4 Department of Radiology, The First Affiliated Hospital of Zhejiang Chinese Medical University, Hangzhou, 310003 Zhejiang China; 5 Department of Gastroenterology, The First People’s Hospital of Huzhou, The First Affiliated Hospital of Huzhou Teachers College, Huzhou, 313000 Zhejiang China

**Keywords:** Bifico, Gut microbiota, Fecal metabolites, Inflammatory cytokines

## Abstract

**Purpose:**

Gut microbiota dysbiosis, a core pathophysiology of irritable bowel syndrome (IBS), is closely related to immunological and metabolic functions. Gut microbiota-based therapeutics have been recently explored in several studies. Bifico is a probiotic cocktail widely used in gastrointestinal disorders which relate to the imbalance of gut microbiota. However, the efficacy and potential mechanisms of Bifico treatment in IBS remains incompletely understood.

**Methods:**

Adopting a wrap restraint stress (WRS) -induced IBS mice model. Protective effect of Bifico in IBS mice was examined through abdominal withdrawal reflex (AWR) scores. 16S rDNA, ^1^H nuclear magnetic resonance (^1^H-NMR) and western blot assays were performed to analyze alterations of gut microbiota, microbiome metabolites and inflammatory cytokines, respectively.

**Results:**

Bifico could decrease intestinal visceral hypersensitivity. Although gut microbiota diversity did not increase, composition of gut microbiota was changed after treatment of Bifico, which were characterized by an increase of *Proteobacteria* phylum and *Actinobacteria* phylum, *Muribaculum* genus, *Bifidobacterium* genus and a decrease of *Parabacteroides* genus, *Sutterella* genus and *Lactobacillus* genus. Moreover, Bifico elevated the concentration of short-chain fatty acids (SCFAs) and reduced protein levels of interleukin-6 (IL-6) and tumor necrosis factor-α (TNF-α). From further Spearman’s correlation analysis, *Bifidobacterium* genus were positively correlated with SCFAs including propionate, butyrate, valerate and negatively correlated with IL-6 and TNF-α.

**Conclusion:**

Bifico could alleviate symptoms of IBS mice through regulation of the gut microbiota, elevating production of SCFAs and reducing the colonic inflammatory response.

**Supplementary Information:**

The online version contains supplementary material available at 10.1007/s00394-022-02958-0.

## Introduction

Irritable bowel syndrome (IBS) is a chronic functional gastrointestinal disorder characterized by recurrent abdominal discomfort and disturbed defecation such as a change in stool frequency or form [1, 2]. Between 5 and 10% of the general population suffers from this condition [3]. However, the underlying etiology and pathogenesis of IBS are incompletely understood. Visceral hypersensitivity, alteration of gut microbiota, chronic inflammation, psychological factors and genetics have been proposed as possible mechanisms in the pathogenesis of IBS [4]. In recent years, increasing evidence suggested that gut microbiota dysbiosis might a core of the pathophysiology of IBS [5, 6].

The gut microbiome has been dominated mainly by bacteria, as over 1000 species and 7000 strains have now been characterized [7]. Further, the gut microbiome is closely related to immunological and metabolic functions by producing a common bacterial metabolite short-chain fatty acids (SCFAs) as mediators [8]. Studies have proven that the gut microbiota dysbiosis could trigger host immune response, damage the intestinal motility and barrier function [9–11]. Furthermore, the composition of gut microbiota has been found significant differences between healthy individuals and IBS patients [12].

Considering the pivotal role of the microbiota in IBS, recent research in IBS treatments has been focused on gut microbiome-based therapeutics. Generally well tolerated, probiotics in IBS have become a relatively successful treatment option [13]. Ford AC et al. made a meta-analysis of 35 randomized controlled trials of probiotics including *Lactobacillus*, *Bifidobacterium*, *Streptococcus* and combination probiotics, involving 3452 patients suffering from IBS. They found that probiotics were effective for the treatment IBS [14]. However, it should be noted that not all probiotic formulations are of benefit in IBS patients [15]. *S. boulardii* and Probiotic mixtures containing *Lactobacillus paracasei ssp paracasei F19, Lactobacillus acidophilus La5* and *Bifidobacterium Bb12* both failed to alleviate symptoms of IBS in randomized clinical trials [16, 17]. Therefore, treatment strategies of probiotics should be further defined.

In 2002, Bifico was approved as an over-the-counter (OTC) drug by the Chinese regulatory authority, the State Food and Drug Administration (SFDA), which contains 1.0 × 10^9^ cfu/g *Bifidobacterium,* 1.0 × 10^9^ cfu/g *Lactobacillus acidophilus* and 1.0 × 10^9^ cfu/g *Enterococcus faecalis* [18–20]. As a mixture of viable bacteria, its regulatory functions on the gut microbiota and anti-inflammatory effects on gastrointestinal disorders have been repeatedly confirmed. We previously reported a prospective, randomized, controlled study of treatment of Bifico in antibiotic-induced gut dysbiosis (AIGD) and found that Bifico could not only stabilize microbiota disorders but also ameliorated colon inflammatory reactions [21]. Prophylactic therapy with Bifico could also reduce the occurrence of neonatal nosocomial enteric infection (NNEI) and decrease the relapse of ulcerative colitis (UC) [19, 22]. Using experimental colitis mice, Bifico was found to ameliorate gut inflammation by decreasing the tumor necrosis factor-α (TNF-α) level [23]. In a study on chronic functional diarrhea, Bifico was able to reduce drug withdrawal in patients compared to the control group [24]. However, there was no relevant research to illustrate the efficacy of Bifico in IBS and its potential mechanisms.

To solve these issues, we adopted a wrap restraint stress (WRS)—induced IBS mice model. As the classical model which was introduced more than 30 years ago for human IBS [25], it represented a suitable model for reproducing the main symptoms present in IBS including visceral hypersensitivity [26–28]. Abdominal withdrawal reflex (AWR) scores to examine the treatment effect of Bifico, following by 16S rDNA gene sequencing to assess the alterations of the gut microbiome, ^1^H nuclear magnetic resonance (^1^H-NMR) to evaluate differential metabolites of fecal samples and western blot assays to detect changes of inflammatory cytokines. Finally, we performed Spearman’s correlation analysis to find relationships among the gut microbiome of fecal samples, metabolites and inflammatory cytokines.

## Materials and methods

### Animals

Seven-week-old male C57BL/6 mice were purchased from the Laboratory Animal Center of Zhejiang Chinese Medical University, Hangzhou, China. All mice were housed in metal barred cages (5 mice/cage) and under controlled conditions (22 ± 1 °C, 55 ± 10% humidity, low noise) with a 12 h light/dark cycle. Water and food were provided ad libitum.

**Fig. 1:**
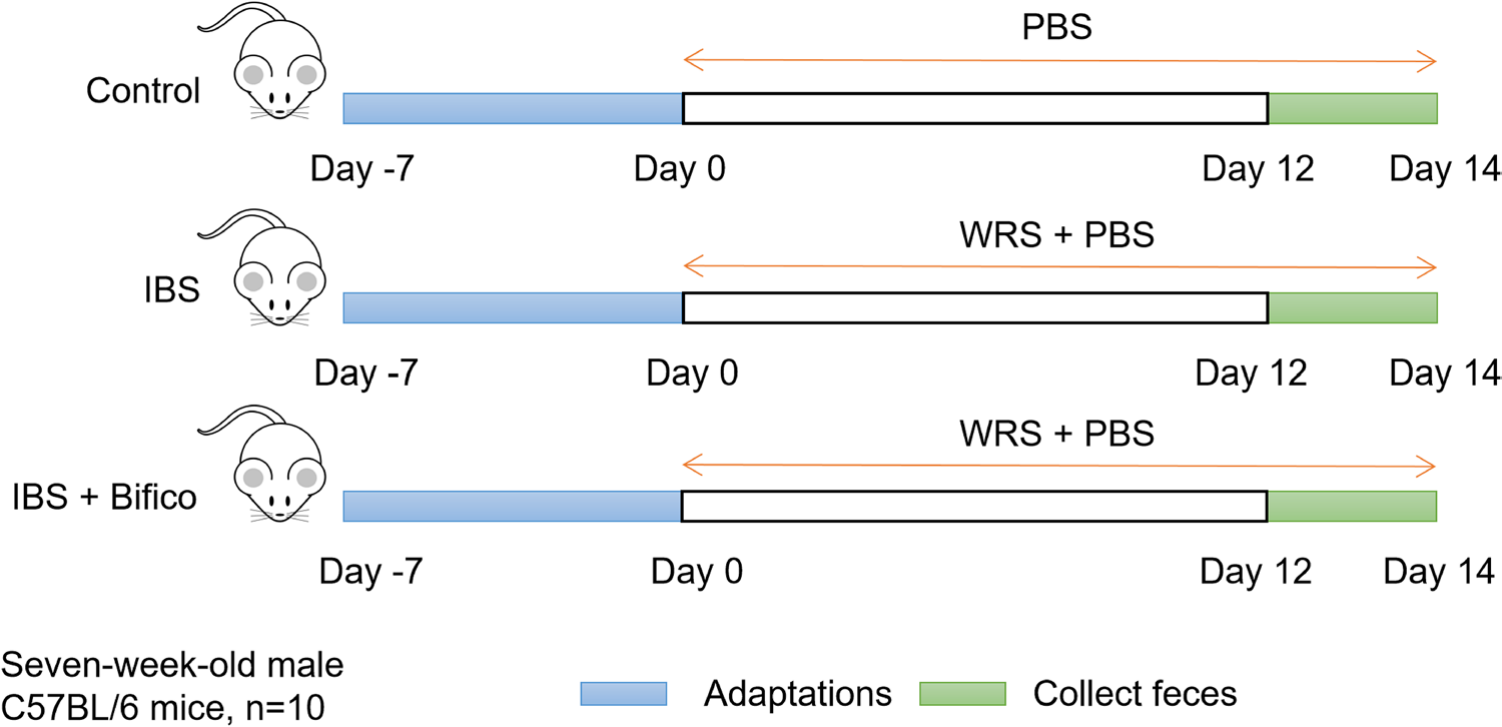
Schematic illustrations of experimental protocols. Intervention timeline for the control group, IBS group, and IBS + Bifico group. *IBS* irritable bowel syndrome, *WRS* wrap restraint stress, *PBS* phosphate buffer saline

After adaptive feeding for 7 days, the mice were randomly divided into 3 groups (*n* = 10/group): control group, IBS group and IBS + Bifico group. The control group were given an intragastric administration of phosphate buffer saline (PBS) (10 ml/kg) once a day. The IBS group and the IBS + Bifico group were induced by WRS procedures. Subsequently, the IBS group was given an intragastric administration of PBS (10 ml/kg) once a day and the IBS + Bifico group was given an intragastric administration of Bifico (Shanghai Sinepharm, Shanghai, China) (0.78 g/kg) once a day. Six mice in each group were randomly selected for biological experiments and sacrificed by CO_2_ inhalation [29]. Body weights of mice were recorded daily. Fresh fecal samples were collected from mice on the last two days and stored at − 80 °C for further analyses. The experimental workflow was shown in (Fig. 1). Experimental protocols conformed to the requirements of the Experimental Animal Ethical Committee of the Zhejiang Chinese Medical University (No. ZSLL-2018–014).

### Wrap-restraint stress model

Stress was induced using a WRS procedure, an acute non-ulcerogenic model of restrain. All the stress sessions were performed between 8 and 10 am for 14 days. During forcing immobilization, they were placed in 50 mL tubes with a small hole for air and cotton ball were used to fill the extra space as described previously [26].

### AWR test: visceral hypersensitivity evaluation

Visceral sensitivity was evaluated at the end of each experiment as follows [30, 31]. A disposable silicon balloon-urethral catheter for pediatric use (6 Fr, Terumo, Tokyo, Japan) was inserted into the rectum to apply colorectal distension (CRD). The balloon was placed 2 cm distal from the anus. After insertion, CRD stimulation was maintained at three different levels of distention (0.25, 0.35, 0.50 mL, respectively) via water injection. Each distention was repeated 3 times, with an interval of 4 min. Average values of AWR scores were calculated as the final score for each mouse. The scoring of the AWR was quantified as previously described [32]. 0 = no behavioral response to distension; 1 = brief head movements followed by immobility; 2 = contraction of abdominal muscles without lifting of the abdomen; 3 = lifting of the abdomen; 4 = body arching and lifting of the pelvic structure.

### Fecal samples preparation for 16S rDNA sequencing

Fresh fecal samples were collected from mice on the last two days. DNA from different samples (at least 200 mg for each sample) was extracted using the E.Z.N.A. ^®^Stool DNA Kit (D4015, Omega, Inc., USA) according to the manufacturer’s instructions. The V3-V4 region of the bacterial 16S rRNA gene was amplified with primers 341F (5ʹ-CCTACGGGNGGCWGCAG-3ʹ) and 805R (5ʹ-GACTACHVGGGTATCTAATCC-3ʹ) [33]. The polymerase chain reaction (PCR) products were purified by AMPure XT beads (Beckman Coulter Genomics, Danvers, MA, USA) and quantified by Qubit (Invitrogen, USA). The 5ʹ ends of the primers were tagged with specific sequencing universal primers. PCR amplification was performed in a total volume of 25 μL reaction mixture containing 25 ng of template DNA, 12.5 μL PCR Premix, 2.5 μL of each primer, and PCR-grade water to adjust the volume. The PCR conditions to amplify the prokaryotic 16S fragments consisted of an initial denaturation at 98 ℃ for 30 s; 32 cycles of denaturation at 98 ℃ for 10 s, annealing at 54 ℃ for 30 s, and extension at 72 ℃ for 45 s; and then final extension at 72 ℃ for 10 min. The PCR products were confirmed with 2% agarose gel electrophoresis. Throughout the DNA extraction process, ultrapure water was used as a negative control to exclude the possibility of false-positive PCR results. The PCR products were purified by AMPure XT beads (Beckman Coulter Genomics, Danvers, MA, USA) and quantified by Qubit (Invitrogen, USA) [34].

### 16S rDNA sequencing and data analysis

The amplicon pools were prepared for sequencing and the size and quantity of the amplicon library were assessed on an Agilent 2100 Bioanalyzer (Agilent, USA) and with the Library Quantification Kit for Illumina (Kapa Biosciences, Woburn, MA, USA), respectively. The libraries were sequenced on NovaSeq PE250 platform. Samples were sequenced on an Illumina NovaSeq platform according to the manufacturer’s recommendations, provided by LC-Bio. Paired-end reads were assigned to samples based on their unique barcode and truncated by cutting off the barcode and primer sequence. Paired-end reads were merged using FLASH. Quality filtering on the raw reads was performed under specific filtering conditions to obtain the high-quality clean tags according to the fqtrim (v0.94). Chimeric sequences were filtered using Vsearch software (v2.3.4). After dereplication using DADA2, we obtained a feature table and a feature sequence.

Alpha diversity and beta diversity were calculated by QIIME2, in which the same number of sequences were extracted randomly through reducing the number of sequences to the minimum of some samples, and the relative abundance (X bacteria count/total count) was used in bacteria taxonomy. Pictures of Alpha diversity and Beta diversity were drawn by R (v3.5.2). The sequence alignment of species annotation was performed by Blast, and the alignment database used was the SILVA and NT-16S [35].

### Fecal samples preparation for ^1^H-NMR analysis

The method of fecal sample preparation was described in a previous study [36]. Briefly, 100 mg thawed stool material were mixed with 0.8 mL PBS containing 10% deuterated water (D2O 99.8%; SIGMA, United States) and 0.05 mM sodium 3-trimethylsilyl-propionate-d4 (TMSP-2,2,3,3-d4; SIGMA, Untied States) as a chemical shift reference. The mixture was immersed into ice for 30 min and then dissolved for 10 cycles (one cycle includes 20 s ultrasound, 10 s crash, and 30 s rest). Then the fecal slurry was centrifuged at 13,000*g* for 10 min at 4 ℃ for twice to obtained supernatants.

### ^1^H-NMR analysis and data processing

The method of ^1^H-NMR analysis and data processing were described in a previous study [37]. Briefly, all ^1^H-NMR spectra were recorded by Bruker 600 MHz AVANCE III spectrometer equipped with a 5 mm-BBFO probe at 25 °C. Shimming and proton pulse calibration was performed automatically for each sample before data acquisition. ^1^H-NMR spectra were received using NOESYPR 1D pulse sequence with water suppression. Bruker Topspin 3.2 was used to process the data.

Free induction decays (FIDs) from ^1^H-NMR of the fecal samples were multiplied by a 0.3 Hz exponential line broadening prior to Fourier Transformation. All NMR spectra were manually phased, baseline corrected and referenced to TSP (δ = 0.0) within MestReNova 12 (Mestrelab Research SL, Spain). The integral region of the spectrum was set between 0.0 and 9.0 ppm, with a spectral region of 4.5–5.0 ppm to eliminate the effects of imperfect water suppression. Due to the deviation of metabolite concentration in the fecal samples of each mouse, each bucket was internally normalized to the total sum of the spectral integrals prior to pattern recognition analysis. The characteristic peaks of all fecal metabolites were determined based on related literature [38, 39] and the Biological Magnetic Resonance Bank (http://www.bmrb.wisc.edu/metabolomics) and Human Metabolome Database (http://www.hmdb.ca/).

### Western blot analysis

Protein extracts were prepared with RIPA Lysis and Extraction Buffer (89,901, Thermo Scientific, USA) supplemented with Protease and Phosphatase Inhibitor Cocktail (78,443, Thermo Scientific, USA) according to the manual. Then proteins were separated on SDS-PAGE gels (10%) followed by transfer to polyvinyl difluoride (PVDF) membranes (pore size 0.2 µm, 88,520, Thermo Scientific, USA). The membrane was subsequently blotted in 1% bovine serum albumin (BSA, Sigma-Aldrich St. Louis, MO, USA) in PBS for 2 h and incubated overnight with commercially available primary antibodies against β-actin (1:1000 dilution, 4970S, Cell Signaling Technology, Danvers, MA, USA), Interleukin-6 (IL-6) (1:1000 dilution, 4970S, Cell Signaling Technology, Danvers, MA, USA) and TNF-a (1:2000 dilution, 41,504, Signalway Antibody, Pearland, TX, USA) at 4 °C. After washing three times with PBS containing 0.05% Tween-20, membranes were incubated with secondary antibodies coupled with HRP (1:4000 dilution, LF102, EpiZyme, Shanghai, China) followed by washing three times. The images were captured with Bio-Rad gel imaging system and analyzed by Quantity One software.

### Statistical analysis

The experimental data were processed and analyzed using Graphpad Prism 6 software (version 6.01). The principal component analysis (PCA) and partial least-squares discriminant analysis (PLS-DA) were performed by SIMCA software, version 14. The non-metric multidimensional scaling (NMDS) analysis and classification tree heat map were made using R language (R version 3.5.2). Venn analysis was pictured by the Bioinformatics website system (http://bioinformatics.psb.ugent.be/webtools/Venn/). Spearman’s correlation analysis was generated by IBM SPSS Statistics 25.0 software. Data was analyzed to perform normality. The Unpaired Student’s two-tailed *t*-test was used for two sets of data conformed to the normal distribution. The Kruskal–Wallis test was used for two sets of data and did not conform to the normal distribution. Analysis of variance (ANOVA) was used to compare multiple groups of data. All values were expressed as mean ± SEM and *P* < *0.05* was considered as statistically significant. Differential metabolomics data must conform to *P* < *0.05* and *VIP* > *1* at the same time.

## Results

### Bifico alleviated visceral hypersensitivity in IBS mice

**Fig. 2:**
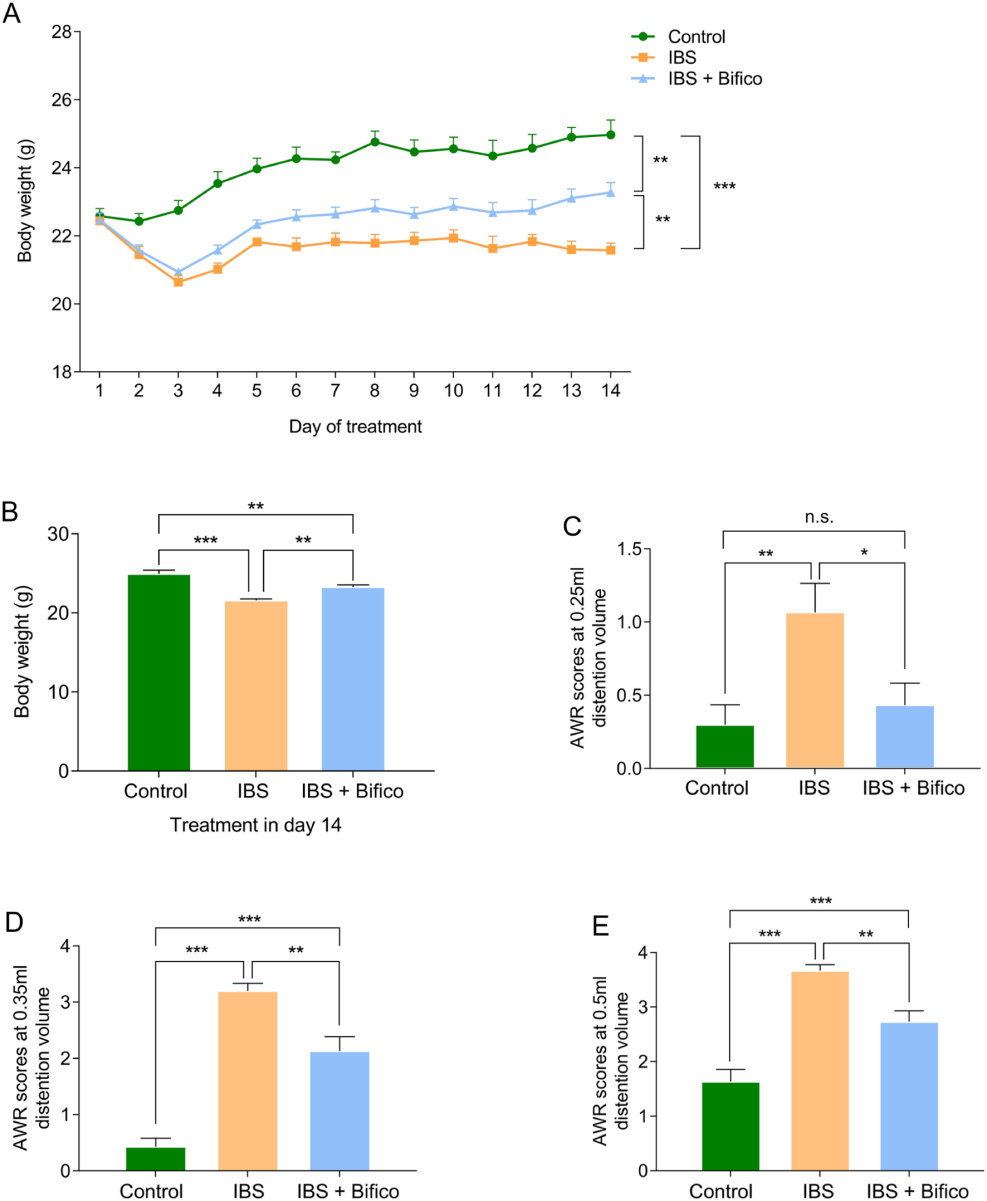
Evaluation of treatment efficacy in IBS mice (*n* = 10/group). **A** Body weight of mice during experiments; **B** Body weight of mice at day 14; AWR scores at a pressure stimulation of 0.25 mL (**C**), 0.35 mL (**D**) and 0.5 mL (**E**). Values were means ± SEM. n.s. represents no significance, *P < 0.05, **P < 0.01, ***P < 0.001

We adopted a WRS model to simulate symptoms of IBS. During the administration of Bifico, changes in body weights were recorded daily. The control group, IBS group and IBS + Bifico group weighted 24.03 ± 0.23, 21.65 ± 0.11 and 22.45 ± 0.17 (mean ± SEM), respectively, which meant that body weights of IBS mice were lower than control mice (*P* < 0.001). Despite after treatment with Bifico, body weights of IBS + Bifico mice were still lower than control mice (*P* < 0.01) they were heavier compared to IBS mice (*P* < 0.01) at the end of the experiment (Fig. 2A, B). To evaluate the development of colonic visceral hypersensitivity, we compared the AWR score at a pressure stimulation of 0.25, 0.35, or 0.5 mL among three groups. The AWR score of the IBS group was significantly higher than the control group (*P* < 0.01 at 0.25 ml, *P* < 0.001 at 0.35 ml and *P* < 0.001 at 0.5 ml, respectively). After Bifico treatment, the AWR score under stimulation with 0.25, 0.35, or 0.5 ml of the Bifico group had significant decreases compared to the IBS group (*P* < 0.05 at 0.25 ml, *P* < 0.01 at 0.35 ml and *P* < 0.01 at 0.5 ml, respectively). Although the AWR score in the Bifico group under stimulation with 0.35 ml and 0.5 ml compared to the control group still higher than in the control group (both *P* < *0.01*), it had no statistical difference compared to the control group under stimulation with 0.25 ml (*P* > 0.05) (Fig. 2C–E). This information suggested that treatment with Bifico could alleviate visceral hypersensitivity in IBS mice.

### Bifico altered the gut microbiota community in IBS mice

**Fig. 3:**
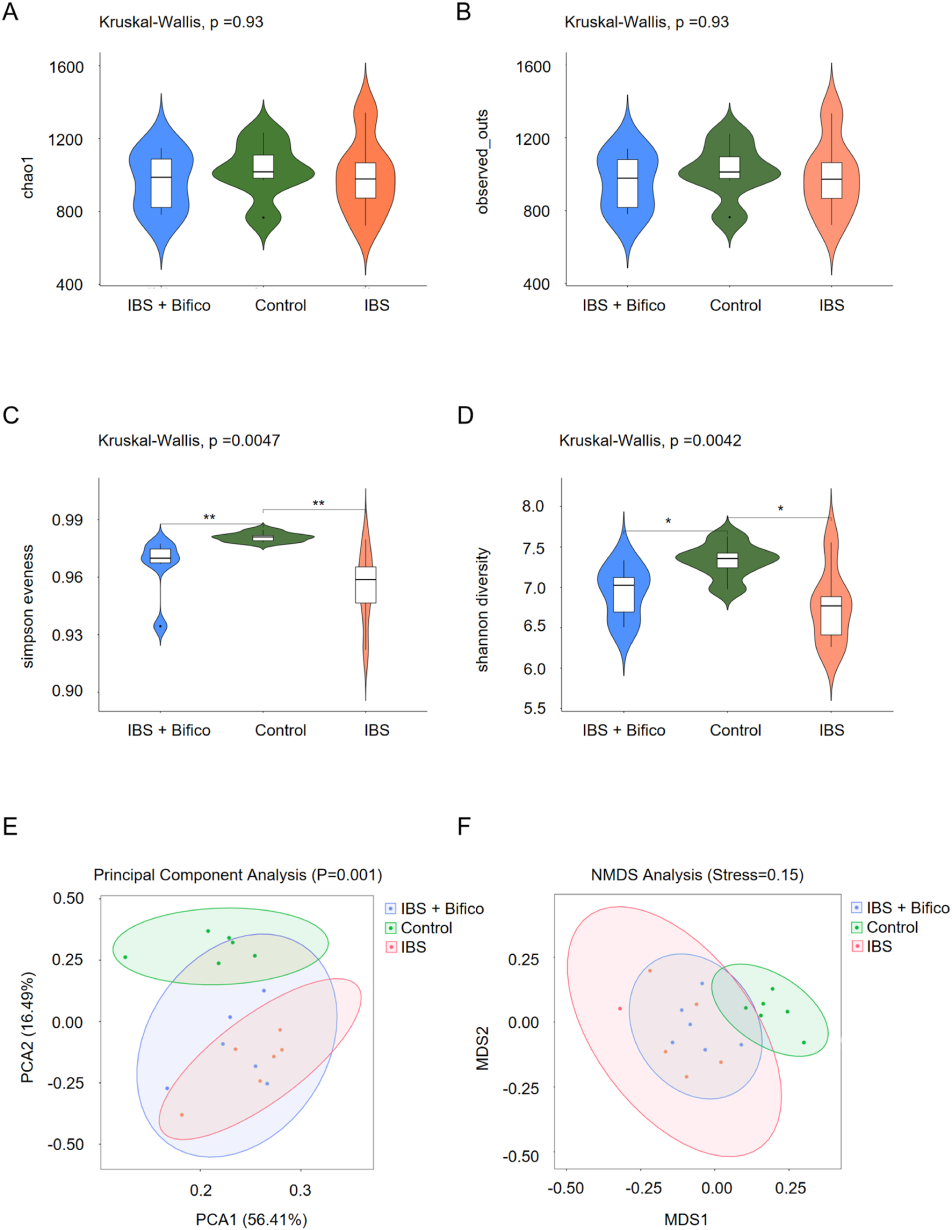
The alpha diversity and beta diversity of gut microbiota in three groups (*n* = 6/group). Alpha diversity of Chao1 (**A**) and Observed_otus (**B**), Simpson Evenness (**C**) and Shannon diversity (**D**); Beta diversity of PCA (**E**) and NMDS (**F**). *PCA* principal component analysis, *NMDS* non-metric multidimensional scaling. *P < 0.05, **P < 0.01

Fecal samples were obtained from mice at the end of treatment. Alpha diversity (Fig. 3A–D) was used to assess the richness and diversity of gut microbiota. Although Chao1 and Observed_otus both showed no difference between the control group and the IBS group ((both Kruskal–Wallis, *P* > 0.05), Simpson Evenness and Shannon diversity of the control group were both higher compared to the IBS group (Simpson Evenness: Kruskal–Wallis, *P* < 0.01 and Shannon diversity: Kruskal–Wallis, *P* < 0.05, respectively). After Bifico treatment, Chao1 and Observed_otus of the Bifico group both showed no difference, Simpson Evenness and Shannon diversity both still lower in the Bifico group compared to the control group (Chao1: Kruskal–Wallis, *P* > 0.05, Observed_otus: Kruskal–Wallis, *P* > 0.05, Simpson Evenness: Kruskal–Wallis, *P* < 0.01 and Shannon diversity: Kruskal–Wallis, *P* < 0.05, respectively), which meant the IBS group was characterized by a diversity reduction and Bifico treatments might not increase gut microbial diversity.

PCA and NMDS of beta diversity (Fig. 3E, F) further revealed significant differences of the gut microbiota community composition between the control group and the IBS group. After Bifico treatment, the community composition of the IBS + Bifico group was closer to the control group. These results indicated that Bifico treatments may affected the microbial community of IBS mice.

**Fig. 4:**
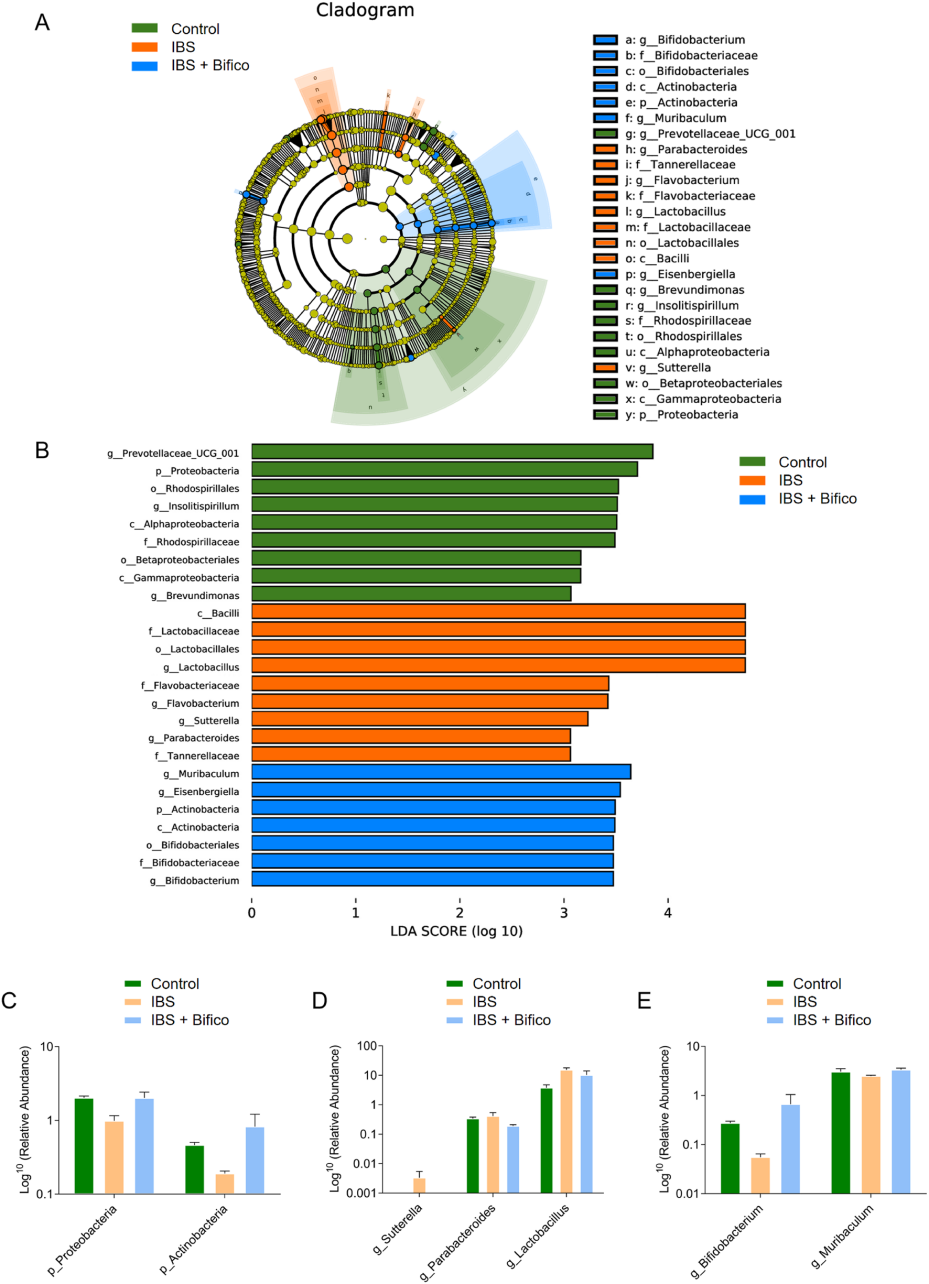
Microbiome structure in three groups (*n* = 6/group). **A** A Cladogram generated by LEfSe analysis; **B** LDA of the gut microbiota; Relative abundances of bacterial phyla level (**C**) and genera level (**D**–**E**). *LDA score (log 10)* > *3* and *P* < 0.05 were considered as significant differences

We further investigated the gut microbiota species and their relative abundance through LEfSe (*LDA score (log 10)* > *3*, *P* < 0.05). When the control group, IBS group and IBS + Bifico group were compared (Fig. 4A, B), 25 phylotypes were identified as key markers of distinct gut microbiota. Their relative values were summarized in Table S1. We focused on phylum and genus levels with opposite trends among the three groups. In our results, *Proteobacteria* was predominant in the control group, while *Actinobacteria* were enriched in the IBS + Bifico group (Fig. 4A, B). Both of them had reduced relative abundance in IBS mice compared to control mice and administration of Bifico could elevate their relative abundance (Fig. 4C). In the general level, the control group was characterized by *Prevotellaceae_UCG_001, Insolitispirillum* and *Brevundimonas*. However, *Lactobacillus, Flavobacterium, Sutterella* and *Parabacteroides* were specific for the IBS group. After administration of Bifico, the IBS + Bifico group was characterized by *Muribaculum, Eisenbergiella* and *Bifidobacterium* (Fig. 4A, B). Among representative microbiota, *Prevotellaceae_UCG_001, Insolitispirillum*, *Brevundimonas* had decreasing trends in the IBS group compared to the control group and *Eisenbergiella* showed an increasing trend in the IBS group compared to the control group. Treatment of Bifico could aggravate these trends (data shown in supplementary Fig. S1), as (Fig. 4C, D) showed, and the relative abundance of *Muribaculum* and *Bifidobacterium* genera were lower in the IBS group than in the control group, while *Lactobacillus, Parabacteroides* and *Sutterella* genera were higher in the IBS group than in the control group. According to the treatment of Bifico, *Muribaculum* and *Bifidobacterium* genera had an increased relative abundance compared to the IBS group, *Lactobacillus, Parabacteroides* and *Sutterella* genera had a decreased relative abundance compared to the IBS group.

### Bifico changed the gut metabolites in IBS mice

**Fig. 5:**
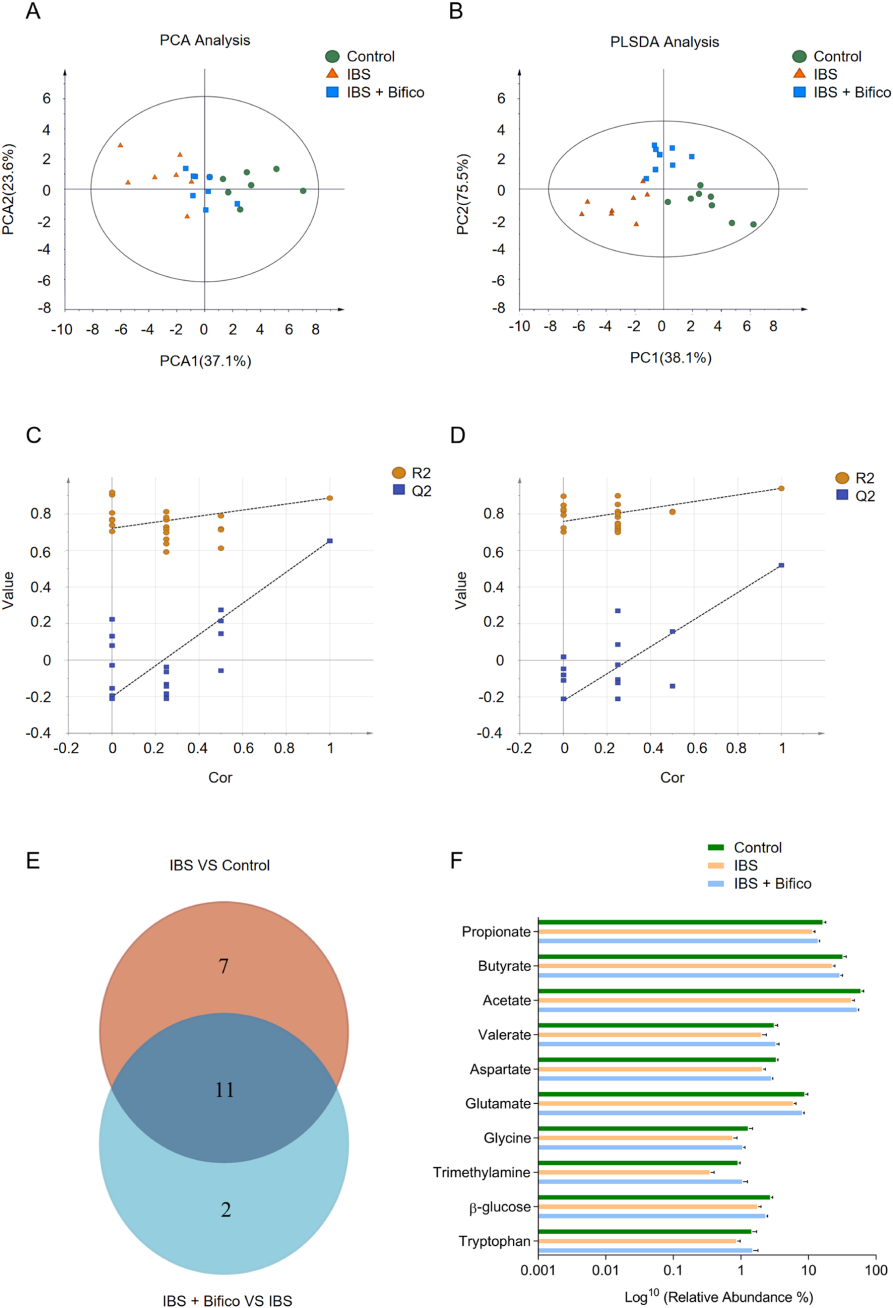
Analyses of fecal metabolites in three groups (*n* = 8/group). PCA **A** and PLS-DA **B** results of fecal metabolites among three groups; Validation plot based on the 1H-NMR spectra of fecal samples observed from the IBS group vs. the Control group **C** and the IBS + Bifico group vs. the IBS group **D**, respectively; **E** Venn diagrams showed the number of altered metabolites between the IBS group and the Control group (orange), the IBS + Bifico group vs. the IBS group (light blue) and their shared metabolites (navy blue); **F** Differential metabolites filtered by variable influence on VIP selection according to the PLS-DA. The filtering conditions *VIP* > *1* and *P* < 0.05. *PLS-DA* partial least-squares discriminant analysis

We next explored the potential changes in metabolites related to the gut microbiota. Different enrichment of metabolites from fecal samples among three groups were observed by ^1^H-NMR spectroscopy. PCA showed that the samples from each group were separated from the other two groups, remarkably, the metabolites of the Bifico group were closer to the metabolites of the control group (Fig. 5A). Moreover, there was a clear distinction among the three groups in the partial PLS-DA, indicating that there were significant differences in the fecal metabolites among the three groups (Fig. 5B). We next verified the credibility and stability of the model. The model parameters were as shown follows: the IBS group vs. the Control group: R2Y = 0.721,* Q*^2^ = − 0.202; the IBS + Bifico group vs. the IBS group: R2Y = 0.759,* Q*^2^ = − 0.222 (Fig. 5C, D), which suggested that the models were stable and accurately predictive.

Then we adopted the criteria of *VIP* > *1* at multivariate statistical analysis and *P* < 0.05 at univariate statistics at the same time for screening differential metabolites among groups. Under the criteria, 18 out of 39 differential metabolites were selected out when the control group was compared to the IBS group. Meanwhile, 13 out of 39 differential metabolites were found to be similar between the IBS group and the IBS + Bifico group. Furthermore, a Venn diagram was used to address the overlapping metabolites among the two collections of differential metabolites (18 and 13, respectively), which marked out 11 metabolites (Fig. 5E). We expected the relative abundance of choline to be lower in the IBS group compared to the control group. It was worth noting that it still had a decreased relative abundance after Bifico treatment compared to the IBS group (data shown in supplementary Fig. S2). There were 10 metabolites including propionate, butyrate, acetate, valerate, aspartate, glutamate, glycine, trimethylamine, β-glucose and tryptophan, which showed a decreased tendency in the IBS group compared to the control group, an elevated tendency in the IBS + Bifico group compared to the IBS group (Fig. 5F), which elucidated that IBS affected the production of gut metabolites, and some of them were reversed by Bifico administration.

### Bifico reduced the expression of TNF-ɑ and IL-6 in IBS mice

**Fig. 6:**
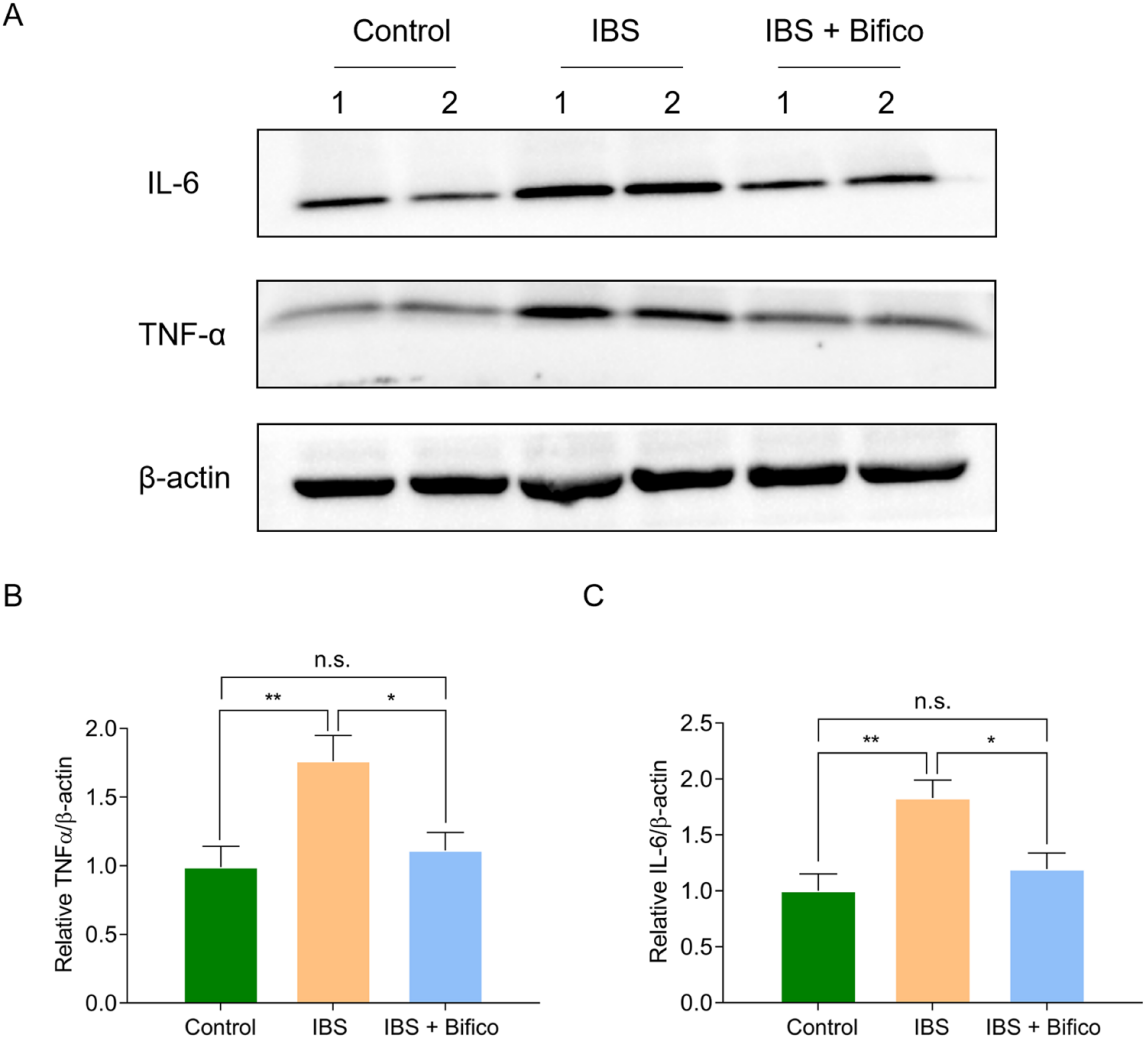
The levels of IL-6 and TNF-ɑ protein levels in mice colon (*n* = 6/group). Quantification of IL-6 (**A**, **B**) and TNF-ɑ (**A**, **C**) expression as determined by western blot analysis, normalized to β-actin expression. Values were means ± SEM. *n.s.* represents no significance, *P < 0.05, **P < 0.01, ***P < 0.001

Because gut microbial dysbiosis is often accompanied by abnormal expression of inflammatory cytokines[40], we evaluated the protein levels of TNF-ɑ and IL-6 in colon tissues and confirmed that expression of TNF-ɑ and IL-6 increased in the IBS group compared with the control group (TNF-ɑ: *P* < 0.01 and IL-6: *P* < 0.01, respectively). However, treatment of Bifico could restore protein expression to normal levels (TNF-ɑ: the IBS group vs. the IBS + Bifico group: *P* < 0.05, the control group vs. the IBS + Bifico group: *P* > 0.05, respectively and IL-6: the IBS group vs. the IBS + Bifico group: *P* < 0.05, the control group vs. the IBS + Bifico group: *P* > 0.05, respectively) (Fig. 6), which indicated that Bifico treatment relieved the colonic inflammation in IBS mice.

### *Bifidobacterium* genera might be main contributor in the treatment of Bifico

**Fig. 7:**
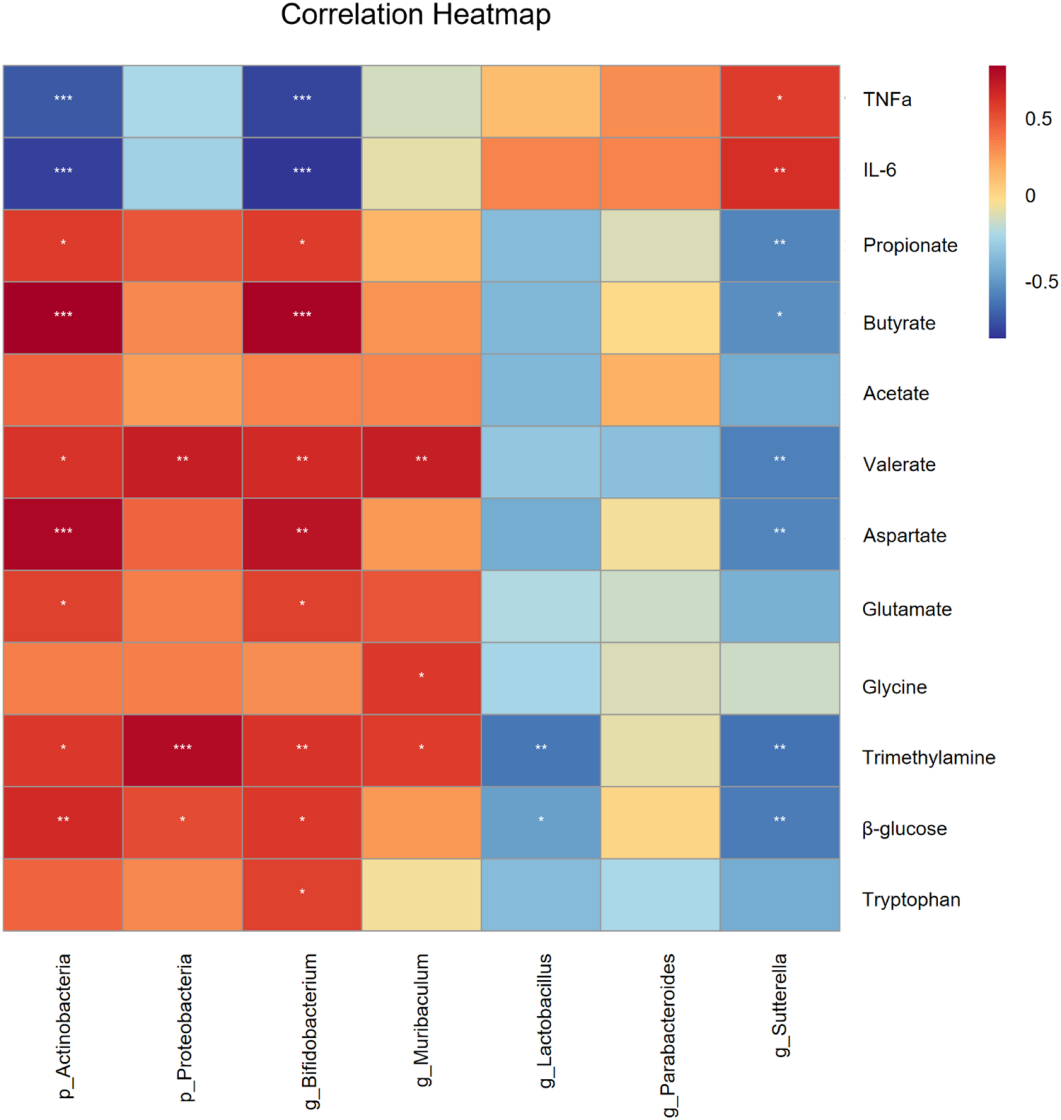
Heat map of the relative abundances of differential gut microbiota, fecal metabolites and inflammatory cytokines identified among groups. Each row represents metabolites or inflammatory cytokines. Each column represents gut microbiota in the phyla level or the genera level. Red represents that the gut microbiota was positively correlated with metabolites or inflammation. Blue color represents that the gut microbiota was negatively correlated with metabolites or inflammation. *P < 0.05, **P < 0.01, ***P < 0.001

For a better understanding of the relationship of gut microbiota, fecal metabolites and inflammatory cytokines which were significantly different among the three groups were analyzed. A heatmap was calculated by the Spearman’s correlation index (Fig. 7). Involving in inflammatory cytokines, we observed that *Actinobacteria* phylum and *Bifidobacterium* genus (belongs to *Actinobacteria* phylum) were negatively correlated with IL-6 and TNF-ɑ, whereas *Sutterella* (belongs to *Proteobacteria* phylum) genus was positively correlated with IL-6 and TNF-ɑ. As for metabolites, *Bifidobacterium* genus were positively correlated with propionate, butyrate, valerate, aspartate, glutamate, trimethylamine, β-glucose and tryptophan. Dramatically, although *Proteobacteria* phylum were positively correlated with valerate, trimethylamine and β-glucose, *Sutterella* genus were negatively correlated with propionate, butyrate, valerate, aspartate, trimethylamine and β-glucose. In addition, *Muribaculum* genus were positively correlated with glycine, trimethylamine and valerate. *Lactobacillus* genus were negatively correlated with β-glucose and trimethylamine. *Parabacteroides* genus were positively correlated with choline. These results revealed that pro-inflammatory factors had a significant positive correlation with *Sutterella* genus and a significant negative correlation with *Bifidobacterium* genus, SCFAs had a significant positive correlation with *Bifidobacterium* and *Muribaculum* genera, and a significant negative correlation with *Sutterella* genus. Further, the *Bifidobacterium* genus might be main contributor in the treatment of Bifico.

## Discussion

IBS is a functional gastrointestinal disorder characterized by visceral hypersensitivity, intestinal immune activation and gut microbiota dysfunction [41]. Recently, a growing body of evidence has suggested that gut microbiome plays a pivotal role in colonic inflammation [42]. As a probiotic mixture, Bifico is supplied for the treatment of microbiota disorders or alleviating the inflammatory reaction [43, 44]. However, how Bifico treatment functions in IBS is still unclear. Here we analyzed the relationship between the gut microbiota and the inflammatory cytokines in IBS after treatment with Bifico, which might provide a theoretical basis for the clinical use of Bifico. Our studies showed that Bifico may relieve the symptoms of IBS by reducing the protein expression level of IL-6 and TNF-α, altering fecal metabolites and gut microbiota. Further studies revealed that *Bifidobacterium* genera may play important roles in treatment.

Previous studies have demonstrated that *Bifidobacterium* and *Lactobacillus* could specifically relieve the symptoms of IBS [45]. Moreover, multispecies probiotics containing strains of more than one genus show enhanced effects in treating antibiotic-associated diarrhea in children, which suggests that probiotic mixtures may more be effective than a single-strain [46]. A meta-analysis evaluated effect of probiotic supplementation on symptoms of patients with IBS. In 11 randomized controlled trials, three studies used a mono-strain probiotic, whereas the remaining eight trials used multi-strain probiotic. Overall, the beneficial effect was more distinct in trials which intervention 8 weeks or longer with multi-strain supplementations [47]. As a type of multispecies probiotic, during treatment of Bifico, both *Lactobacillus acidophilus* and *Enterococcus faecalis* might promote proliferation of *Bifidobacterium*. Previous research evidenced that *Lactobacillus acidophilus* could produce bifidogenic growth factors to stimulate the growth of *Bifidobacterium longum* in pure culture [48]*. Enterococcus faecalis* could create anaerobic conditions, which might be of benefit for survival of *Bifidobacterium* [49]. In addition, *Enterococcus* spp. has been used as a probiotic to defend against gut infection and prevent the colonization of more pathogenic bacteria [50].

WRS is an accepted method of creating an acute stress-induced IBS model [26]. In this study, we observed that treatment of Bifico significantly alleviated intestinal visceral hypersensitivity and reduced weight loss. These results were consistent with most of the previous studies that effective treatment could reduce the AWR score of IBS [51–53]. Since IBS often involves gut microbiome dysbiosis, we collected fecal samples to analyze the gut microbiota. Overall differences of gut microbiota were often assessed through alpha diversity and beta diversity. As for alpha diversity, community richness was measured by Chao1 and Observed_otus, while community diversity was measured by Simpson Evenness and Shannon diversity. It should be noted that Trends of alpha diversity had been controversial. Despite in some previous studies, comparing to healthy controls, alpha diversity of gut microbiota in IBS patients or IBS mouse models were significantly lower, some studies showed alpha diversity were increased or not significantly changed [54–58]. Beta diversity included PCA and PCoA analyses, both of them were used to assess differences in gut microbiota composition. Most findings supported that the beta diversity of gut microbiota in IBS patients or mouse models were significantly different from healthy controls [59–61]. In this research, results showed that Chao1 and Observed_otus of the IBS group had no significant difference compared to the control group, but Simpson Evenness and Shannon diversity was lower in the IBS group than in the control group, these results were similar to previous findings by Fukui H. et al. [62]. However, treatment of Bifico did not alter alpha diversity of IBS. In beta diversity, the bacterial composition of the IBS + Bifico group was closer to the control group through treatment of Bifico, which consisted with previous research findings. To further analyze the gut microbiota and their relative abundance according to LEfSe. Because of differences in subtype, region, design scheme, sample size, etc., the change trend of gut microbiota in IBS was heterogeneous. Meta-analysis pointed out that comparing participants with IBS to healthy controls, higher relative abundance of pro-inflammatory bacteria, lower *Bifidobacterium* and *Lactobacillus* was observed [59, 63]. But it should be noticed that some researchers hold different views of *Lactobacillus.* Clinical research from Japan observed that IBS patients had significantly higher counts of *Lactobacillus* [64]. Another clinical study considered that Lactobacillus may have no effect on IBS patients [65]. In this research, the relative abundance of *Proteobacteria* and *Actinobacteria* phyla were markedly lower in the IBS group than in the control group and the IBS + Bifico group. In the genera level, *Parabacteroides* and *Sutterella* were increased in the IBS group compared to the control group and the IBS + Bifico group. *Parabacteroides and Parabacteroides merdae* (belongs to *Parabacteroides* genus) were considered as potentially pathogenic bacterium that were reported to be frequently enriched in the hypertensive gut microbiome [66, 67]. Moreover, in infectious diseases*, Parabacteroides merdae* is generally considered an opportunistic pathogen, which is able to develop antimicrobial drug resistance [68]. *Sutterella* is a controversial bacterium. From a previous review, *Sutterella* was related to better outcomes in patients with IBD [69]. Berer K et al. held the opinion that *Sutterella* had anti-inflammatory functions in vitro [70]. But some studies have suspected *Sutterella* plays a role in the disease progression of IBD [71]. Furthermore, in clinical studies, no difference was observed in the prevalence of *Sutterella* spp. between the IBD patients and the healthy subjects [72, 73]. Surprisingly, *Lactobacillus* expressed higher levels in the IBS group than in the control group and the IBS + Bifico group. Compared to the control group and the IBS + Bifico group, the IBS group showed a significant decrease in the abundance of *Muribaculum* and *Bifidobacterium.* Yuan Y et al. noted that reduction of *Muribaculum* could result in inflammation, dyslipidemia and glucose intolerance [74]. As for *Bifidobacterium*, which belongs to *Actinobacteria* phylum, numerous studies have shown that it had benefits in improving epithelial barrier function in mice, acting as an anti-inflammatory agent and a source of SCFAs [75, 76]. In addition, in dextran sulfate sodium-induced colitis mice, *Bifidobacterium* not only downregulated levels of IL-6 and TNF-α, but also upregulated level of IL-10 [77].

SCFAs describe acetic acid, propionic acid, butyric acid, valeric acid and caproic acid, which are the main byproducts of gut metabolites [78]. In the colon, the proportion of acetate, propionate, and butyrate can reach 90–95% of SCFAs [79]. It has been widely reported that SCFAs play pivotal roles in anti-inflammatory and maintenance of intestinal health such as locomotion recovery [80–82]. SCFAs included higher amounts of acetate, propionate, butyrate and lower amounts of formate, valerate, and caproate [83]. Butyrate as one of main SCFAs, could attenuate visceral hypersensitivity of IBS mice and increased Interleukin-10 (IL-10) production [84, 85]. In addition, butyrate and propionate could promote peripheral regulatory T cell generation of mice [86]. Through enhancing IL-10 production and suppressing Th17 cells, valerate also might be of therapeutic relevance for inflammatory diseases [87]. What’s more, SCFAs are involved in lipid metabolism and glucose metabolism [88]. According to the altered gut bacteria, we speculate that colonic metabolites may have changed. Through ^1^H NMR spectroscopy, we found that the abundance of acetate, propionate, butyrate and valerate were decreased in the IBS group, which supported the view of suggesting that patients with IBS had lower levels of SCFAs [89]. Treatment of Bifico could increase their abundance. From Spearman’s correlation analysis, we found that *Actinobacteria* phylum and *Bifidobacterium* genus were positively correlated with propionate, butyrate and valerate, which were consistent with previous report [90]. *Proteobacteria* phylum and *Muribaculum* genus were positively correlated with valerate. The *Sutterella* genus were negatively correlated with propionate, butyrate and valerate.

Changes in the gut microbiota can induce or aggravate inflammation [91]. Recent research regards TNF-α as a vital inflammatory cytokine in IBS [92]. In addition, IL-6 has reproducibly been detected to be elevated in IBS patients and rats [93–95]. In our studies, the protein level in colonic IL-6 and TNF-α were higher in the IBS group than in the control group, further confirmed by the previous results. Zhao HM et al. found that in the colon of mice with colitis, the level of TNF-α could be significantly reduced by Bifico [96]. Our results supported the that Bifico could reduce both the protein level of IL-6 and TNF-α in the colon, alleviating the inflammation to a certain extent. In further correlation analysis, *Actinobacteria* phylum and *Bifidobacterium* genera were negatively correlated with IL-6 and TNF-α, verifying the anti-inflammatory function reported by Chichlowski M et al. [97]. Interestingly, *Sutterella* genera were positively correlated with IL-6 and TNF-α. This result supported that the *Sutterella* genera had a pro-inflammatory capacity in the human gastrointestinal tract by Hiippala K et al. [98].

Taken together, using a widely developed IBS mice model, we found that treatment of Bifico could decrease intestinal visceral hypersensitivity of IBS mice. This effect might through improved gut microbiota disorders such as increase relative abundance of *Bifidobacterium* and *Muribaculum* genera and decrease relative abundance of *Sutterella* genus to elevate levels of SCFAs or reduce levels of pro-inflammatory cytokines. In the clinical treatment of IBS, probiotics are widely used in managements of gut microbiota disorders [99]. However, because of varieties of strains and combinations, which particular combination, species or strains of probiotics are effective for IBS remains unclear. From recent meta-analysis, the beneficial effects were more distinct in the trials using multi-strain supplements [47]. Future research may be addressed to confirm the improvement effect of Bifico on the gut microbiota of IBS patients and compare with mono-strain supplement.

## Supplementary Information

Below is the link to the electronic supplementary material.

Supplementary file1 Supplementary Fig. 1 Changes in gut microbiota. (A) The relative abundance of Eisenbergiella genera. (B) The relative abundance of Insolitispirillum genera, Brevundimonas genera and Prevotellaceae_UCG_001 genera. LDA score (log 10) > 3, P < 0.05 (TIF 198003 KB)

Supplementary file2 Fig. 2 Changes of choline in fecal metabolites. VIP > 1, P < 0.05 (TIF 197251 KB)

Supplementary file3 (DOC 83 KB)

## Abbreviations

IBS: Irritable bowel syndrome
AWR: Abdominal withdrawal reflex
1H-NMR: 1H nuclear magnetic resonance
SCFAs: Short-chain fatty acids
IL-6: Interleukin-6
TNF-α: Tumor necrosis factor-α
OTC: Over-the-counter
SFDA: State food and drug administration
AIGD: Antibiotic-induced gut dysbiosis
NNEI: Neonatal nosocomial enteric infection
UC: Ulcerative colitis
WRS: Wrap restraint stress
PBS: Phosphate buffer saline
CRD: Colorectal distension
PCR: Polymerase chain reaction
D2O: Deuterated water
FIDs: Free induction decays
PVDF: Polyvinyl difluoride
PCA: Principal component
PLS-DA: Partial least-squares discriminant analysis
NMDS: Analysis non-metric multidimensional scaling
ANOVA: Analysis of variance
LEfSe: Linear discriminant analysis effect size
IL-10: Interleukin-10

## References

[1] Villani AC, Lemire M, Thabane M, Belisle A, Geneau G, Garg AX, Clark WF, Moayyedi P, Collins SM, Franchimont D, Marshall JK (2010). Genetic risk factors for post-infectious irritable bowel syndrome following a waterborne outbreak of gastroenteritis. Gastroenterology.

[2] Chen YJ, Wu H, Wu SD, Lu N, Wang YT, Liu HN, Dong L, Liu TT, Shen XZ (2018). Parasutterella, in association with irritable bowel syndrome and intestinal chronic inflammation. J Gastroenterol Hepatol.

[3] Ford AC, Sperber AD, Corsetti M, Camilleri M (2020). Irritable bowel syndrome. Lancet.

[4] Shukla R, Ghoshal U, Ranjan P, Ghoshal UC (2018). Expression of toll-like receptors, pro-, and anti-inflammatory cytokines in relation to gut microbiota in irritable bowel syndrome: the evidence for its micro-organic basis. J Neurogastroenterol Motil.

[5] Pimentel M, Lembo A (2020). Microbiome and its role in irritable bowel syndrome. Dig Dis Sci.

[6] Singh R, Zogg H, Wei L, Bartlett A, Ghoshal UC, Rajender S, Ro S (2021). Gut microbial dysbiosis in the pathogenesis of gastrointestinal dysmotility and metabolic disorders. J Neurogastroenterol Motil.

[7] Pusceddu MM, Gareau MG (2018). Visceral pain: gut microbiota, a new hope?. J Biomed Sci.

[8] Levy M, Blacher E, Elinav E (2017). Microbiome, metabolites and host immunity. Curr Opin Microbiol.

[9] Cani PD (2016). Gut microbiota: changes in gut microbes and host metabolism: squaring the circle?. Nat Rev Gastroenterol Hepatol.

[10] Bhattarai Y, Muniz Pedrogo DA, Kashyap PC (2017). Irritable bowel syndrome: a gut microbiota-related disorder?. Am J Physiol Gastrointest Liver Physiol.

[11] Sciavilla P, Strati F, Di Paola M, Modesto M, Vitali F, Cavalieri D, Prati GM, Di Vito M, Aragona G, De Filippo C, Mattarelli P (2021). Gut microbiota profiles and characterization of cultivable fungal isolates in IBS patients. Appl Microbiol Biotechnol.

[12] Tap J, Derrien M, Tornblom H, Brazeilles R, Cools-Portier S, Dore J, Storsrud S, Le Neve B, Ohman L, Simren M (2017). Identification of an intestinal microbiota signature associated with severity of irritable bowel syndrome. Gastroenterology.

[13] Pretorius L, Smith C (2020). The trace aminergic system: a gender-sensitive therapeutic target for IBS?. J Biomed Sci.

[14] Ford AC, Quigley EM, Lacy BE, Lembo AJ, Saito YA, Schiller LR, Soffer EE, Spiegel BM, Moayyedi P (2014). Efficacy of prebiotics, probiotics, and synbiotics in irritable bowel syndrome and chronic idiopathic constipation: systematic review and meta-analysis. Am J Gastroenterol.

[15] Ghoshal UC, Gwee KA, Holtmann G, Li Y, Park SJ, Simadibrata M, Sugano K, Wu K, Quigley EMM, Cohen H (2018). The role of the microbiome and the use of probiotics in gastrointestinal disorders in adults in the Asia-Pacific region - background and recommendations of a regional consensus meeting. J Gastroenterol Hepatol.

[16] Choi CH, Jo SY, Park HJ, Chang SK, Byeon JS, Myung SJ (2011). A randomized, double-blind, placebo-controlled multicenter trial of saccharomyces boulardii in irritable bowel syndrome: effect on quality of life. J Clin Gastroenterol.

[17] Begtrup LM, de Muckadell OB, Kjeldsen J, Christensen RD, Jarbol DE (2013). Long-term treatment with probiotics in primary care patients with irritable bowel syndrome–a randomised, double-blind, placebo controlled trial. Scand J Gastroenterol.

[18] Yu H, Liu L, Chang Z, Wang S, Wen B, Yin P, Liu D, Chen B, Zhang J (2013). Genome Sequence of the bacterium Bifidobacterium longum strain CMCC P0001, a probiotic strain used for treating gastrointestinal disease. Genome Announc.

[19] Huang NN, Wang GZ., Wang JF, Yuan YX (2016) Risk factors for neonatal nosocomial enteric infection and the effect of intervention with BIFICO. Eur Rev Med Pharmacol Sci 20(17): 3713–3719. https://www.ncbi.nlm.nih.gov/pubmed/27649676 27649676

[20] Shi CZ, Chen HQ, Liang Y, Xia Y, Yang YZ, Yang J, Zhang JD, Wang SH, Liu J, Qin HL (2014). Combined probiotic bacteria promotes intestinal epithelial barrier function in interleukin-10-gene-deficient mice. World J Gastroenterol.

[21] Wu J, Gan T, Zhang Y, Xia G, Deng S, Lv X, Zhang B, Lv B (2020). The prophylactic effects of BIFICO on the antibiotic-induced gut dysbiosis and gut microbiota. Gut Pathog.

[22] Cui HH, Chen CL, Wang JD, Yang YJ, Cun Y, Wu JB, Liu YH, Dan HL, Jian YT, Chen XQ (2004). Effects of probiotic on intestinal mucosa of patients with ulcerative colitis. World J Gastroenterol.

[23] Zhang Y, Zhao X, Zhu Y, Ma J, Ma H, Zhang H (2018). Probiotic mixture protects dextran sulfate sodium-induced colitis by altering tight junction protein expressions and increasing tregs. Mediators Inflamm.

[24] Yao-Zong Y, Shi-Rong L, Delvaux M (2004). Comparative efficacy of dioctahedral smectite (Smecta) and a probiotic preparation in chronic functional diarrhoea. Dig Liver Dis.

[25] Williams CL, Villar RG, Peterson JM, Burks TF (1988). Stress-induced changes in intestinal transit in the rat: a model for irritable bowel syndrome. Gastroenterology.

[26] Lin L, Feng B, Zhou R, Liu Y, Li L, Wang K, Yu Y, Liu C, Long X, Gu X, Li B, Wang X, Yang X, Cong Y, Zuo X, Li Y (2020). Acute stress disrupts intestinal homeostasis via GDNF-RET. Cell Prolif.

[27] Traini C, Evangelista S, Girod V, Faussone-Pellegrini MS, Vannucchi MG (2016). Changes of excitatory and inhibitory neurotransmitters in the colon of rats underwent to the wrap partial restraint stress. Neurogastroenterol Motil.

[28] Taguchi R, Shikata K, Furuya Y, Hirakawa T, Ino M, Shin K, Shibata H (2017). Selective corticotropin-releasing factor 1 receptor antagonist E2508 reduces restraint stress-induced defecation and visceral pain in rat models. Psychoneuroendocrinology.

[29] Hong KB, Seo H, Lee JS, Park Y (2019). Effects of probiotic supplementation on post-infectious irritable bowel syndrome in rodent model. BMC Complement Altern Med.

[30] Zhao Q, Yang WR, Wang XH, Li GQ, Xu LQ, Cui X, Liu Y, Zuo XL (2019). Clostridium butyricum alleviates intestinal low-grade inflammation in TNBS-induced irritable bowel syndrome in mice by regulating functional status of lamina propria dendritic cells. World J Gastroenterol.

[31] Al-Chaer ED, Kawasaki M, Pasricha PJ (2000). A new model of chronic visceral hypersensitivity in adult rats induced by colon irritation during postnatal development. Gastroenterology.

[32] Wang FY, Su M, Zheng YQ, Wang XG, Kang N, Chen T, Zhu EL, Bian ZX, Tang XD (2015). Herbal prescription Chang'an II repairs intestinal mucosal barrier in rats with post-inflammation irritable bowel syndrome. Acta Pharmacol Sin.

[33] Logue JB, Stedmon CA, Kellerman AM, Nielsen NJ, Andersson AF, Laudon H, Lindstrom ES, Kritzberg ES (2016). Experimental insights into the importance of aquatic bacterial community composition to the degradation of dissolved organic matter. ISME J.

[34] Zhang B, Chen T, Cao M, Yuan C, Reiter RJ, Zhao Z, Zhao Y, Chen L, Fan W, Wang X, Zhou X, Li C (2022). Gut Microbiota dysbiosis induced by decreasing endogenous melatonin mediates the pathogenesis of Alzheimer’s disease and obesity. Front Immunol.

[35] Feng P, Li Q, Liu L, Wang S, Wu Z, Tao Y, Huang P, Wang P (2022). Crocetin prolongs recovery period of DSS-induced colitis via altering intestinal microbiome and increasing intestinal permeability. Int J Mol Sci.

[36] Chen H, Zhang F, Zhang J, Zhang X, Guo Y, Yao Q (2020). A holistic view of berberine inhibiting intestinal carcinogenesis in conventional mice based on microbiome-metabolomics analysis. Front Immunol.

[37] Chen H, Zhang F, Li R, Liu Y, Wang X, Zhang X, Xu C, Li Y, Guo Y, Yao Q (2020). Berberine regulates fecal metabolites to ameliorate 5-fluorouracil induced intestinal mucositis through modulating gut microbiota. Biomed Pharmacother.

[38] Swann JR, Garcia-Perez I, Braniste V, Wilson ID, Sidaway JE, Nicholson JK, Pettersson S, Holmes E (2017). Application of (1)H NMR spectroscopy to the metabolic phenotyping of rodent brain extracts: a metabonomic study of gut microbial influence on host brain metabolism. J Pharm Biomed Anal.

[39] Xu M, Ye J, Yang D, Xu X, Yeo TT, Ng WH, Lim CC (2007). Ex-vivo NMR of unprocessed tissue in water: a simplified procedure for studying intracranial neoplasms. Anal Bioanal Chem.

[40] Li Q, Li L, Li Q, Wang J, Nie S, Xie M (2022). Foods.

[41] Li J, Cui H, Cai Y, Lin J, Song X, Zhou Z, Xiong W, Zhou H, Bian Y, Wang L (2018). Tong-Xie-Yao-Fang regulates 5-HT level in diarrhea predominant irritable bowel syndrome through gut microbiota modulation. Front Pharmacol.

[42] Seksik P, Sokol H, Lepage P, Vasquez N, Manichanh C, Mangin I, Pochart P, Dore J, Marteau P (2006). Review article: the role of bacteria in onset and perpetuation of inflammatory bowel disease. Aliment Pharmacol Ther.

[43] Yu HJ, Liu W, Chang Z, Shen H, He LJ, Wang SS, Liu L, Jiang YY, Xu GT, An MM, Zhang JD (2015). Probiotic BIFICO cocktail ameliorates Helicobacter pylori induced gastritis. World J Gastroenterol.

[44] Jiang XE, Yang SM, Zhou XJ, Zhang Y (2020). Effects of mesalazine combined with bifid triple viable on intestinal flora, immunoglobulin and levels of cal, MMP-9, and MPO in feces of patients with ulcerative colitis. Eur Rev Med Pharmacol Sci.

[45] Brenner DM, Moeller MJ, Chey WD, Schoenfeld PS (2009). The utility of probiotics in the treatment of irritable bowel syndrome: a systematic review. Am J Gastroenterol.

[46] Timmerman HM, Koning CJ, Mulder L, Rombouts FM, Beynen AC (2004). Monostrain, multistrain and multispecies probiotics–A comparison of functionality and efficacy. Int J Food Microbiol.

[47] Dale HF, Rasmussen SH, Asiller OO, Lied GA (2019). Probiotics in irritable bowel syndrome: an up-to-date systematic review. Nutrients.

[48] Warda AK, Clooney AG, Ryan F, de Almeida Bettio PH, Di Benedetto G, Ross RP, Hill C (2021). A postbiotic consisting of heat-treated lactobacilli has a bifidogenic effect in pure culture and in human fermented faecal communities. Appl Environ Microbiol.

[49] Ya'ari S, Halperin-Sternfeld M, Rosin B, Adler-Abramovich L (2020). Surface modification by nano-structures reduces viable bacterial biofilm in aerobic and anaerobic environments. Int J Mol Sci.

[50] Kabwe M, Meehan-Andrews T, Ku H, Petrovski S, Batinovic S, Chan HT, Tucci J (2021). Lytic bacteriophage EFA1 modulates HCT116 colon cancer cell growth and upregulates ROS production in an enterococcus faecalis co-culture system. Front Microbiol.

[51] Song YF, Pei LX, Chen L, Geng H, Yuan MQ, Xu WL, Wu J, Zhou JY, Sun JH (2020). Electroacupuncture relieves irritable bowel syndrome by regulating IL-18 and gut microbial dysbiosis in a trinitrobenzene sulfonic acid-induced post-inflammatory animal model. Am J Chin Med.

[52] Zhang Y, Zhang H, Zhang W, Zhang Y, Wang W, Nie L (2020). LncRNA XIST modulates 5-hydroxytrytophan-induced visceral hypersensitivity by epigenetic silencing of the SERT gene in mice with diarrhea-predominant IBS. Cell Signal.

[53] Chen BR, Du LJ, He HQ, Kim JJ, Zhao Y, Zhang YW, Luo L, Dai N (2017). Fructo-oligosaccharide intensifies visceral hypersensitivity and intestinal inflammation in a stress-induced irritable bowel syndrome mouse model. World J Gastroenterol.

[54] Phan J, Nair D, Jain S, Montagne T, Flores DV, Nguyen A, Dietsche S, Gombar S, Cotter P (2021). Alterations in gut microbiome composition and function in irritable bowel syndrome and increased probiotic abundance with daily supplementation. mSystems.

[55] Barandouzi ZA, Lee J, Maas K, Starkweather AR, Cong XS (2021). Altered gut microbiota in irritable bowel syndrome and its association with food components. J Pers Med.

[56] Tramullas M, Collins JM, Fitzgerald P, Dinan TG, OM SM, Cryan JF (2021). Estrous cycle and ovariectomy-induced changes in visceral pain are microbiota-dependent. Science.

[57] Chen Q, Ren Y, Lu J, Bartlett M, Chen L, Zhang Y, Guo X, Liu C (2017). A novel prebiotic blend product prevents irritable bowel syndrome in mice by improving gut microbiota and modulating immune response. Nutrients.

[58] Barandouzi ZA, Lee J, Del Carmen Rosas M, Chen J, Henderson WA, Starkweather AR, Cong XS (2022). Associations of neurotransmitters and the gut microbiome with emotional distress in mixed type of irritable bowel syndrome. Sci Rep.

[59] Zhuang X, Xiong L, Li L, Li M, Chen M (2017). Alterations of gut microbiota in patients with irritable bowel syndrome: a systematic review and meta-analysis. J Gastroenterol Hepatol.

[60] Song H, Wang W, Shen B, Jia H, Hou Z, Chen P, Sun Y (2018). Pretreatment with probiotic Bifico ameliorates colitis-associated cancer in mice: Transcriptome and gut flora profiling. Cancer Sci.

[61] Lo Presti A, Del Chierico F, Altomare A, Zorzi F, Cella E, Putignani L, Guarino MPL, Monteleone G, Cicala M, Angeletti S, Ciccozzi M (2019). Exploring the genetic diversity of the 16S rRNA gene of Akkermansia muciniphila in IBD and IBS. Future Microbiol.

[62] Fukui H, Nishida A, Matsuda S, Kira F, Watanabe S, Kuriyama M, Kawakami K, Aikawa Y, Oda N, Arai K, Matsunaga A, Nonaka M, Nakai K, Shinmura W, Matsumoto M, Morishita S, Takeda AK, Miwa H (2020). Usefulness of machine learning-based gut microbiome analysis for identifying patients with irritable bowels syndrome. J Clin Med.

[63] Wang L, Alammar N, Singh R, Nanavati J, Song Y, Chaudhary R, Mullin GE (2020). Gut microbial dysbiosis in the irritable bowel syndrome: a systematic review and meta-analysis of case-control studies. J Acad Nutr Diet.

[64] Tana C, Umesaki Y, Imaoka A, Handa T, Kanazawa M, Fukudo S (2010). Altered profiles of intestinal microbiota and organic acids may be the origin of symptoms in irritable bowel syndrome. Neurogastroenterol Motil.

[65] O'Mahony L, McCarthy J, Kelly P, Hurley G, Luo F, Chen K, O'Sullivan GC, Kiely B, Collins JK, Shanahan F, Quigley EM (2005). Lactobacillus and bifidobacterium in irritable bowel syndrome: symptom responses and relationship to cytokine profiles. Gastroenterology.

[66] Hu X, Li H, Zhao X, Zhou R, Liu H, Sun Y, Fan Y, Shi Y, Qiao S, Liu S, Liu H, Zhang S (2021). Multi-omics study reveals that statin therapy is associated with restoration of gut microbiota homeostasis and improvement in outcomes in patients with acute coronary syndrome. Theranostics.

[67] Yan Q, Gu Y, Li X, Yang W, Jia L, Chen C, Han X, Huang Y, Zhao L, Li P, Fang Z, Zhou J, Guan X, Ding Y, Wang S, Khan M, Xin Y, Li S, Ma Y (2017). Alterations of the gut microbiome in hypertension. Front Cell Infect Microbiol.

[68] Boente RF, Ferreira LQ, Falcao LS, Miranda KR, Guimaraes PL, Santos-Filho J, Vieira JM, Barroso DE, Emond JP, Ferreira EO, Paula GR, Domingues RM (2010). Detection of resistance genes and susceptibility patterns in Bacteroides and Parabacteroides strains. Anaerobe.

[69] Morgan BP, Harris CL (2015). Complement, a target for therapy in inflammatory and degenerative diseases. Nat Rev Drug Discov.

[70] Berer K, Gerdes LA, Cekanaviciute E, Jia X, Xiao L, Xia Z, Liu C, Klotz L, Stauffer U, Baranzini SE, Kumpfel T, Hohlfeld R, Krishnamoorthy G, Wekerle H (2017). Gut microbiota from multiple sclerosis patients enables spontaneous autoimmune encephalomyelitis in mice. Proc Natl Acad Sci U S A.

[71] Lavelle A, Lennon G, O'Sullivan O, Docherty N, Balfe A, Maguire A, Mulcahy HE, Doherty G, O'Donoghue D, Hyland J, Ross RP, Coffey JC, Sheahan K, Cotter PD, Shanahan F, Winter DC, O'Connell PR (2015). Spatial variation of the colonic microbiota in patients with ulcerative colitis and control volunteers. Gut.

[72] Mukhopadhya I, Hansen R, Nicholl CE, Alhaidan YA, Thomson JM, Berry SH, Pattinson C, Stead DA, Russell RK, El-Omar EM, Hold GL (2011). A comprehensive evaluation of colonic mucosal isolates of Sutterella wadsworthensis from inflammatory bowel disease. PLoS ONE.

[73] Hansen R, Berry SH, Mukhopadhya I, Thomson JM, Saunders KA, Nicholl CE, Bisset WM, Loganathan S, Mahdi G, Kastner-Cole D, Barclay AR, Bishop J, Flynn DM, McGrogan P, Russell RK, El-Omar EM, Hold GL (2013). The microaerophilic microbiota of de-novo paediatric inflammatory bowel disease: the BISCUIT study. PLoS ONE.

[74] Yuan Y, Zhou J, Zheng Y, Xu Z, Li Y, Zhou S, Zhang C (2020). Beneficial effects of polysaccharide-rich extracts from Apocynum venetum leaves on hypoglycemic and gut microbiota in type 2 diabetic mice. Biomed Pharmacother.

[75] Rahman S, Davids M, van Hamersveld PHP, Welting O, Rahaoui H, Schuren F, Meijer SL, van den Wijngaard RM, Hakvoort TBM, de Jonge WJ, Heinsbroek SEM (2021). Dietary curdlan enhances bifidobacteria and reduces intestinal inflammation in mice. Nutrients.

[76] de la Cuesta-Zuluaga J, Mueller NT, Corrales-Agudelo V, Velasquez-Mejia EP, Carmona JA, Abad JM, Escobar JS (2017). Metformin is associated with higher relative abundance of mucin-degrading Akkermansia muciniphila and several short-chain fatty acid-producing microbiota in the gut. Diabetes Care.

[77] Chen Y, Chen H, Ding J, Stanton C, Ross RP, Zhao J, Zhang H, Yang B, Chen W (2021). Bifidobacterium longum ameliorates dextran sulfate sodium-induced colitis by producing conjugated linoleic acid, protecting intestinal mechanical barrier, restoring unbalanced gut microbiota, and regulating the toll-like receptor-4/nuclear factor-kappaB signaling pathway. J Agric Food Chem.

[78] Zietek M, Celewicz Z, Szczuko M (2021). Short-chain fatty acids. maternal microbiota and metabolism in pregnancy. Nutrients.

[79] Rios-Covian D, Ruas-Madiedo P, Margolles A, Gueimonde M, de Los Reyes-Gavilan CG, Salazar N (2016). Intestinal short chain fatty acids and their link with diet and human health. Front Microbiol.

[80] McBrearty N, Arzumanyan A, Bichenkov E, Merali S, Merali C, Feitelson M (2021). Short chain fatty acids delay the development of hepatocellular carcinoma in HBx transgenic mice. Neoplasia.

[81] Carretta MD, Quiroga J, Lopez R, Hidalgo MA, Burgos RA (2021). Participation of short-chain fatty acids and their receptors in gut inflammation and colon cancer. Front Physiol.

[82] Jing Y, Yu Y, Bai F, Wang L, Yang D, Zhang C, Qin C, Yang M, Zhang D, Zhu Y, Li J, Chen Z (2021). Effect of fecal microbiota transplantation on neurological restoration in a spinal cord injury mouse model: involvement of brain-gut axis. Microbiome.

[83] Dalile B, Van Oudenhove L, Vervliet B, Verbeke K (2019). The role of short-chain fatty acids in microbiota-gut-brain communication. Nat Rev Gastroenterol Hepatol.

[84] He Y, Tan Y, Zhu J, Wu X, Huang Z, Chen H, Yang M, Chen D (2021). Effect of sodium butyrate regulating IRAK1 (interleukin-1 receptor-associated kinase 1) on visceral hypersensitivity in irritable bowel syndrome and its mechanism. Bioengineered.

[85] Farup PG, Rudi K, Hestad K (2016). Faecal short-chain fatty acids - a diagnostic biomarker for irritable bowel syndrome?. BMC Gastroenterol.

[86] Arpaia N, Campbell C, Fan X, Dikiy S, van der Veeken J, deRoos P, Liu H, Cross JR, Pfeffer K, Coffer PJ, Rudensky AY (2013). Metabolites produced by commensal bacteria promote peripheral regulatory T-cell generation. Nature.

[87] Luu M, Pautz S, Kohl V, Singh R, Romero R, Lucas S, Hofmann J, Raifer H, Vachharajani N, Carrascosa LC, Lamp B, Nist A, Stiewe T, Shaul Y, Adhikary T, Zaiss MM, Lauth M, Steinhoff U, Visekruna A (2019). The short-chain fatty acid pentanoate suppresses autoimmunity by modulating the metabolic-epigenetic crosstalk in lymphocytes. Nat Commun.

[88] He J, Zhang P, Shen L, Niu L, Tan Y, Chen L, Zhao Y, Bai L, Hao X, Li X, Zhang S, Zhu L (2020). Short-chain fatty acids and their association with signalling pathways in inflammation, glucose and lipid metabolism. Int J Mol Sci.

[89] Goll R, Johnsen PH, Hjerde E, Diab J, Valle PC, Hilpusch F, Cavanagh JP (2020). Effects of fecal microbiota transplantation in subjects with irritable bowel syndrome are mirrored by changes in gut microbiome. Gut Microbes.

[90] Hamad I, Cardilli A, Corte-Real BF, Dyczko A, Vangronsveld J, Kleinewietfeld M (2022). High-salt diet induces depletion of lactic acid-producing bacteria in murine gut. Nutrients.

[91] Shi Y, Zhong L, Li Y, Chen Y, Feng S, Wang M, Xia Y, Tang S (2021). Repulsive guidance molecule b deficiency induces gut microbiota dysbiosis and increases the susceptibility to intestinal inflammation in mice. Front Microbiol.

[92] Li Y, Tian X, Li S, Chang L, Sun P, Lu Y, Yu X, Chen S, Wu Z, Xu Z, Kang W (2019). Total polysaccharides of adlay bran (Coix lachryma-jobi L.) improve TNF-alpha induced epithelial barrier dysfunction in Caco-2 cells via inhibition of the inflammatory response. Food Funct.

[93] Scully P, McKernan DP, Keohane J, Groeger D, Shanahan F, Dinan TG, Quigley EM (2010). Plasma cytokine profiles in females with irritable bowel syndrome and extra-intestinal co-morbidity. Am J Gastroenterol.

[94] O'Malley D, Dinan TG, Cryan JF (2011). Altered expression and secretion of colonic interleukin-6 in a stress-sensitive animal model of brain-gut axis dysfunction. J Neuroimmunol.

[95] O'Malley D, Dinan TG, Cryan JF (2012). Interleukin-6 modulates colonic transepithelial ion transport in the stress-sensitive Wistar Kyoto rat. Front Pharmacol.

[96] Zhao HM, Huang XY, Zuo ZQ, Pan QH, Ao MY, Zhou F, Liu HN, Liu ZY, Liu DY (2013). Probiotics increase T regulatory cells and reduce severity of experimental colitis in mice. World J Gastroenterol.

[97] Chichlowski M, Shah N, Wampler JL, Wu SS, Vanderhoof JA (2020). Bifidobacterium longum subspecies infantis (B. infantis) in pediatric nutrition: current state of knowledge. Nutrients.

[98] Hiippala K, Kainulainen V, Kalliomaki M, Arkkila P, Satokari R (2016). Mucosal prevalence and interactions with the epithelium indicate commensalism of Sutterella spp. Front Microbiol.

[99] Wollny T, Daniluk T, Piktel E, Wnorowska U, Buklaha A, Gluszek K, Durnas B, Bucki R (2021). Targeting the gut microbiota to relieve the symptoms of irritable bowel syndrome. Pathogens.

