# Supplementary material for: Bifico relieves irritable bowel syndrome by regulating gut microbiota dysbiosis and inflammatory cytokines
Source: Eur J Nutr. 2022 Aug 2;62(1):139–55. doi: 10.1007/s00394-022-02958-0 (PMC9899748; doi:10.1007/s00394-022-02958-0)
Supplement: Supplementary file 3 — Supplementary file3 (DOC 83 KB) [file 394_2022_2958_MOESM3_ESM.doc]

Table S1 Statistic Results of key markers of distinct gut microbiota

| dominant microbiota | kruskal.test.  p_value | significance | mean | mean_Control | mean_IBS | mean_Bifico | Control1 | Control2 | Control3 | Control4 | Control5 | Control6 | IBS1 | IBS2 | IBS3 | IBS4 | IBS5 | IBS6 | IBS+  Bifico1 | IBS+  Bifico2 | IBS+  Bifico3 | IBS+  Bifico4 | IBS+  Bifico5 | IBS+  Bifico6 |
| --- | --- | --- | --- | --- | --- | --- | --- | --- | --- | --- | --- | --- | --- | --- | --- | --- | --- | --- | --- | --- | --- | --- | --- | --- |
| g__Prevotellaceae_UCG-001 | 0.0131 | yes | 0.7413 | 1.5761 | 0.4922 | 0.1556 | 1.5531 | 2.2794 | 1.7248 | 0.3457 | 1.5518 | 2.0021 | 0.1926 | 0.0925 | 0.1666 | 0.1272 | 0.2001 | 2.1743 | 0.3308 | 0.0733 | 0.2039 | 0.1168 | 0.0049 | 0.2039 |
| p__Proteobacteria | 0.0250 | yes | 1.6727 | 2.0224 | 0.9852 | 2.0104 | 1.7240 | 1.6942 | 2.2475 | 2.4849 | 1.9969 | 1.9871 | 1.7287 | 0.7108 | 1.2095 | 0.9371 | 0.6038 | 0.7212 | 0.8400 | 1.7364 | 1.0981 | 2.4982 | 2.3539 | 3.5360 |
| o__Rhodospirillales | 0.0193 | yes | 0.4146 | 0.7963 | 0.3072 | 0.1404 | 0.4024 | 0.2870 | 1.5973 | 1.4030 | 0.3254 | 0.7626 | 1.1977 | 0.1234 | 0.0542 | 0.3772 | 0.0847 | 0.0060 | 0.0899 | 0.1048 | 0.1587 | 0.1736 | 0.2124 | 0.1027 |
| g__Insolitispirillum | 0.0183 | yes | 0.3542 | 0.7259 | 0.2500 | 0.0867 | 0.3583 | 0.2731 | 1.3917 | 1.3827 | 0.2379 | 0.7115 | 0.9701 | 0.0705 | 0.0444 | 0.3443 | 0.0649 | 0.0060 | 0.0747 | 0.0819 | 0.0918 | 0.1138 | 0.0794 | 0.0785 |
| c__Actinobacteria | 0.0034 | yes | 0.3638 | 0.3047 | 0.0779 | 0.7089 | 0.2122 | 0.2759 | 0.3693 | 0.3298 | 0.3024 | 0.3384 | 0.0700 | 0.0485 | 0.0903 | 0.1018 | 0.0559 | 0.1011 | 2.5733 | 0.4381 | 0.7092 | 0.2111 | 0.1151 | 0.2069 |
| f__Rhodospirillaceae | 0.0250 | yes | 0.3846 | 0.7346 | 0.2895 | 0.1298 | 0.3707 | 0.2801 | 1.4047 | 1.3827 | 0.2471 | 0.7220 | 1.1145 | 0.1131 | 0.0444 | 0.3742 | 0.0847 | 0.0060 | 0.0899 | 0.1048 | 0.1223 | 0.1676 | 0.1945 | 0.0997 |
| o__Betaproteobacteriales | 0.0380 | yes | 0.5096 | 0.6881 | 0.3861 | 0.4544 | 0.8145 | 0.7329 | 0.4055 | 0.6581 | 1.1389 | 0.3791 | 0.3034 | 0.3569 | 0.6360 | 0.3488 | 0.3352 | 0.3365 | 0.3384 | 0.4998 | 0.7325 | 0.4266 | 0.1945 | 0.5347 |
| c__Gammaproteobacteria | 0.0446 | yes | 0.5554 | 0.7158 | 0.4175 | 0.5329 | 0.8351 | 0.7412 | 0.4475 | 0.7000 | 1.1588 | 0.4122 | 0.3195 | 0.4112 | 0.6596 | 0.3727 | 0.3497 | 0.3923 | 0.4756 | 0.5501 | 0.8185 | 0.5044 | 0.2659 | 0.5830 |
| g__Brevundimonas | 0.0345 | yes | 0.0007 | 0.0021 | 0.0000 | 0.0000 | 0.0000 | 0.0070 | 0.0029 | 0.0029 | 0.0000 | 0.0000 | 0.0000 | 0.0000 | 0.0000 | 0.0000 | 0.0000 | 0.0000 | 0.0000 | 0.0000 | 0.0000 | 0.0000 | 0.0000 | 0.0000 |
| c__Bacilli | 0.0298 | yes | 9.8047 | 3.8237 | 15.3056 | 10.2849 | 1.6496 | 6.7769 | 0.6488 | 5.9461 | 3.8157 | 4.1050 | 6.5020 | 17.2612 | 15.0919 | 16.9396 | 25.6083 | 10.4308 | 26.9265 | 9.8715 | 11.7542 | 1.0673 | 10.8876 | 1.2023 |
| f__Lactobacillaceae | 0.0298 | yes | 9.7770 | 3.7883 | 15.2695 | 10.2732 | 1.6441 | 6.7351 | 0.5662 | 5.9229 | 3.7819 | 4.0794 | 6.4582 | 17.2333 | 15.0836 | 16.9067 | 25.5903 | 10.3448 | 26.9235 | 9.8686 | 11.7338 | 1.0523 | 10.8633 | 1.1978 |
| o__Lactobacillales | 0.0298 | yes | 9.8045 | 3.8237 | 15.3049 | 10.2849 | 1.6496 | 6.7769 | 0.6488 | 5.9461 | 3.8157 | 4.1050 | 6.4976 | 17.2612 | 15.0919 | 16.9396 | 25.6083 | 10.4308 | 26.9265 | 9.8715 | 11.7542 | 1.0673 | 10.8876 | 1.2023 |
| g__Lactobacillus | 0.0298 | yes | 9.7657 | 3.7862 | 15.2571 | 10.2539 | 1.6386 | 6.7282 | 0.5662 | 5.9229 | 3.7819 | 4.0794 | 6.4480 | 17.2333 | 15.0586 | 16.8962 | 25.5903 | 10.3161 | 26.9052 | 9.8686 | 11.7090 | 1.0523 | 10.8568 | 1.1313 |
| f__Flavobacteriaceae | 0.0345 | yes | 0.0008 | 0.0000 | 0.0025 | 0.0000 | 0.0000 | 0.0000 | 0.0000 | 0.0000 | 0.0000 | 0.0000 | 0.0029 | 0.0000 | 0.0083 | 0.0000 | 0.0036 | 0.0000 | 0.0000 | 0.0000 | 0.0000 | 0.0000 | 0.0000 | 0.0000 |
| g__Flavobacterium | 0.0345 | yes | 0.0008 | 0.0000 | 0.0025 | 0.0000 | 0.0000 | 0.0000 | 0.0000 | 0.0000 | 0.0000 | 0.0000 | 0.0029 | 0.0000 | 0.0083 | 0.0000 | 0.0036 | 0.0000 | 0.0000 | 0.0000 | 0.0000 | 0.0000 | 0.0000 | 0.0000 |
| g__Sutterella | 0.0085 | yes | 0.0011 | 0.0000 | 0.0034 | 0.0000 | 0.0000 | 0.0000 | 0.0000 | 0.0000 | 0.0000 | 0.0000 | 0.0058 | 0.0044 | 0.0000 | 0.0030 | 0.0072 | 0.0000 | 0.0000 | 0.0000 | 0.0000 | 0.0000 | 0.0000 | 0.0000 |
| g__Parabacteroides | 0.0084 | yes | 0.3149 | 0.3399 | 0.4135 | 0.1914 | 0.2853 | 0.4110 | 0.4953 | 0.3428 | 0.2026 | 0.3023 | 0.5325 | 0.2511 | 0.2319 | 0.2425 | 0.2469 | 0.9762 | 0.2302 | 0.2284 | 0.1296 | 0.1407 | 0.1962 | 0.2235 |
| f__Tannerellaceae | 0.0084 | yes | 0.3149 | 0.3399 | 0.4135 | 0.1914 | 0.2853 | 0.4110 | 0.4953 | 0.3428 | 0.2026 | 0.3023 | 0.5325 | 0.2511 | 0.2319 | 0.2425 | 0.2469 | 0.9762 | 0.2302 | 0.2284 | 0.1296 | 0.1407 | 0.1962 | 0.2235 |
| g__Muribaculum | 0.0338 | yes | 2.9646 | 3.0502 | 2.4958 | 3.3477 | 2.7204 | 2.5386 | 3.0831 | 5.3183 | 2.4374 | 2.2037 | 2.5296 | 2.4730 | 2.4288 | 2.7020 | 2.1178 | 2.7235 | 2.9986 | 4.2944 | 2.7088 | 3.4353 | 3.7400 | 2.9091 |
| g__Eisenbergiella | 0.0470 | yes | 0.3550 | 0.0296 | 0.2328 | 0.8025 | 0.0069 | 0.0167 | 0.0768 | 0.0289 | 0.0184 | 0.0301 | 0.0890 | 0.0426 | 0.0917 | 0.0195 | 0.2848 | 0.8691 | 0.0518 | 0.5429 | 0.0102 | 0.1063 | 1.4363 | 2.6675 |
| p__Actinobacteria | 0.0109 | yes | 0.4924 | 0.4619 | 0.1907 | 0.8248 | 0.3128 | 0.4305 | 0.4938 | 0.6060 | 0.4451 | 0.4829 | 0.1473 | 0.1410 | 0.2027 | 0.2515 | 0.1874 | 0.2143 | 2.7013 | 0.6348 | 0.8607 | 0.3039 | 0.1654 | 0.2825 |
| c__Acidimicrobiia | 0.8737 | no | 0.0010 | 0.0010 | 0.0013 | 0.0008 | 0.0000 | 0.0000 | 0.0000 | 0.0000 | 0.0061 | 0.0000 | 0.0000 | 0.0044 | 0.0000 | 0.0000 | 0.0036 | 0.0000 | 0.0046 | 0.0000 | 0.0000 | 0.0000 | 0.0000 | 0.0000 |
| o__Bifidobacteriales | 0.0063 | yes | 0.3295 | 0.2731 | 0.0525 | 0.6629 | 0.1764 | 0.2118 | 0.3403 | 0.2893 | 0.2947 | 0.3264 | 0.0452 | 0.0279 | 0.0764 | 0.0808 | 0.0559 | 0.0287 | 2.5169 | 0.3964 | 0.6685 | 0.1766 | 0.0648 | 0.1541 |
| f__Bifidobacteriaceae | 0.0063 | yes | 0.3295 | 0.2731 | 0.0525 | 0.6629 | 0.1764 | 0.2118 | 0.3403 | 0.2893 | 0.2947 | 0.3264 | 0.0452 | 0.0279 | 0.0764 | 0.0808 | 0.0559 | 0.0287 | 2.5169 | 0.3964 | 0.6685 | 0.1766 | 0.0648 | 0.1541 |
